# Psychosocial adversity and socioeconomic position during childhood and epigenetic age: analysis of two prospective cohort studies

**DOI:** 10.1093/hmg/ddy036

**Published:** 2018-01-22

**Authors:** Rebecca B Lawn, Emma L Anderson, Matthew Suderman, Andrew J Simpkin, Tom R Gaunt, Andrew E Teschendorff, Martin Widschwendter, Rebecca Hardy, Diana Kuh, Caroline L Relton, Laura D Howe

**Affiliations:** 1 MRC Integrative Epidemiology Unit, University of Bristol, UK; 2 School of Experimental Psychology, University of Bristol, UK; 3 Department of Population Health Sciences, Bristol Medical School, University of Bristol, UK; 4 Department of Women's Cancer, University College London, UK; 5 UCL Cancer Institute, University College London, UK; 6 CAS-Max-Planck Partner Institute for Computational Biology, Shanghai Institute for Biological Sciences, Shanghai 200031, China; 7 MRC Unit for Lifelong Health and Ageing, University College London, UK; 8 Insight Centre for Data Analytics, National University of Ireland, Galway, Ireland

## Abstract

Psychosocial adversity in childhood (e.g. abuse) and low socioeconomic position (SEP) can have significant lasting effects on social and health outcomes. DNA methylation-based biomarkers are highly correlated with chronological age; departures of methylation-predicted age from chronological age can be used to define a measure of age acceleration, which may represent a potential biological mechanism linking environmental exposures to later health outcomes. Using data from two cohorts of women Avon Longitudinal Study of Parents and Children, (ALSPAC), *N = *989 and MRC National Survey of Health and Development, NSHD, *N = *773), we assessed associations of SEP, psychosocial adversity in childhood (parental physical or mental illness or death, parental separation, parental absence, sub-optimal maternal bonding, sexual, emotional and physical abuse and neglect) and a cumulative score of these psychosocial adversity measures, with DNA methylation age acceleration in adulthood (measured in peripheral blood at mean chronological ages 29 and 47 in ALSPAC and buccal cells at age 53 in NSHD). Sexual abuse was strongly associated with age acceleration in ALSPAC (sexual abuse data were not available in NSHD), e.g. at the 47-year time point sexual abuse associated with a 3.41 years higher DNA methylation age (95% CI 1.53 to 5.29) after adjusting for childhood and adulthood SEP. No associations were observed between low SEP, any other psychosocial adversity measure or the cumulative psychosocial adversity score and age acceleration. DNA methylation age acceleration is associated with sexual abuse, suggesting a potential mechanism linking sexual abuse with adverse outcomes. Replication studies with larger sample sizes are warranted.

## Introduction

Psychosocial adversity in childhood (e.g. abuse, neglect, or parental separation) and low socioeconomic position (SEP) can have significant and lasting effects on social, psychological, and health outcomes in later life (1–6). Identifying strategies to reduce or potentially eliminate the harmful effects of early adversity is crucial for combating inequalities. While there is a large body of evidence documenting the long-term consequences of early life psychosocial adversity and SEP, the pathways and mechanisms through which they occur remain unclear. The potential of epigenetics as a set of gene regulatory mechanisms linking environmental exposures to later outcomes is gaining prominence (7,8). DNA methylation, the addition of methyl groups to nucleotide bases, is the epigenetic modification most widely analysed in population studies. Animal models have identified associations between early life stress and altered methylation patterns (9–12); many of these were confirmed in human studies of low SEP(13) and childhood abuse (14–16).

A recent development within epigenetic research is the ‘epigenetic clock'; a set of DNA methylation markers that can be used to estimate chronological age with high accuracy (*R* = 0.96) (17). These estimates are referred to as ‘epigenetic age' and it is said that epigenetic age is accelerated when it is higher than true chronological age (17). One hypothesis is that accelerated epigenetic age indicates accelerated biological aging, potentially attributable to the influence of environmental factors such as stress, diet or disease. Obesity, for example, is known not only to accelerate processes associated with aging (18) but also to accelerate sexual maturation in females (19,20). Not surprisingly, obesity was recently linked to accelerated epigenetic age in the liver (21). Accelerated epigenetic age is associated with lower lung function, cognitive function and physical capability (22), and with increased mortality, with a 5-year difference between chronological and methylation age associated with a 21% higher risk of mortality (23).

In this study, we investigate whether SEP and psychosocial adversity are associated with epigenetic age acceleration, motivated by the idea that this may reflect a biological mechanism linking psychosocial adversity and low SEP in childhood with long-term adverse health outcomes. We used data from two large prospective cohort studies to investigate the associations between psychosocial adversity and SEP in childhood and epigenetic age acceleration from peripheral tissues collected in adulthood. Given the known co-occurrence of multiple forms of psychosocial adversity and the potential presence of cumulative effects on health (24–27), we analyse a cumulative score of psychosocial adversity in addition to exploring whether different types of psychosocial adversity have differing associations with epigenetic age acceleration.

## Results

Data were available for 989 women in the ALSPAC cohort and 773 women in NSHD (Supplementary Material, Tables S1 and S10). The mean age at DNA methylation assessment was 28.7 (SD = 5.54) years for the first time point in ALSPAC, 47.3 (SD = 4.42) years for the second time point in ALSPAC, and 53.4 (SD = 0.16) years in NSHD. The mean derived epigenetic age was similar to mean chronological age for both time points in ALSPAC, but in NSHD epigenetic age was on average 9 years younger than chronological age (Table 1). We found lower than previously reported Pearson correlation coefficients between chronological age and epigenetic age (*r*=0.45, 0.50 and 0.12 for the 29-year ALSPAC, 47-year ALSPAC and NSHD, respectively). Epigenetic age acceleration (the residuals of a regression between chronological age and epigenetic age) was on average close to zero in all samples, with the non-zero mean at the 47-year ALSPAC timepoint being due to the multiple imputation. The correlation between epigenetic age acceleration at the two ALSPAC time points was 0.27.

**Table 1.** Characteristics of participants included in the analysis

Measures of age (years)	ALSPAC		NSHD
	29-year data	49-year data	
	(*n = *989)	(*n = *989)	(*n = *773)
	Mean (SD)		
Chronological age	28.65 (5.54)	47.34 (4.42)	53.44 (0.16)
Epigenetic age	30.05 (6.74)	44.66 (6.86)	42.81 (5.72)
Age acceleration	−0.04 (5.19)	−0.28 (5.51)	−0.02 (5.68)

Table 2 shows the prevalence of low SEP, each type of psychosocial adversity, and each category of the cumulative psychosocial adversity score. The highest prevalence was for maltreatment in ALSPAC and parental physical illness in NSHD. Prevalence was higher in ALSPAC for parental mental illness, parental physical illness, parental separated, parental absence and maltreatment than in NSHD. Child illness, sub-optimal maternal bonding and death of a parent were more common in NSHD than in ALSPAC. Prevalence was slightly higher for manual SEP in childhood within NSHD, with higher levels of cumulative adversity observed in ALSPAC. Associations between different adverse experiences varied (e.g. 5% for parental absence from household and child physical illness, and 46% for parental mental illness and sub-optimal maternal bonding in ALSPAC) (Supplementary Material, Tables S11 and S13). For those who experienced sexual abuse, 6% also experienced physical neglect, and 79% also experienced emotional neglect (Supplementary Material, Table S12).

**Table 2.** Prevalence of low SEP and psychosocial adversity in childhood

	ALSPAC	NSHD	
	(*n = *989)	(*n = *773)
	**% Prevalence**	***P*-value for difference** ^a^
**Manual SEP in childhood**	47.7	56.7	<0.001
**Psychosocial adversity before 17 years:**			
Parent physically ill	28.7	27.2	0.47
Parent absent	16.2	2.3	<0.001
Child illness	5.3	14.1	<0.001
Parent mentally ill	32.5	1.8	<0.001
Sub-optimal maternal bonding	16.5	20.7	0.02
Parents separated	13.3	5.8	<0.001
Parent died	5.1	7.1	0.07
Child maltreatment	23.0	7.2	<0.001
**Cumulative psychosocial adversity score**			
0	33.7	40.0	<0.001
1	28.3	39.2
2	17.0	16.2
3+	21.1	4.5
*Items in maltreatment variable in ALSPAC*			
Physical cruelty	3.5	—	
Emotional cruelty	8.4	—	
Physical neglect	1.5	—	
Emotional neglect	20.2	—	
Sexual abuse	3.8	—	
*Additional items measured only in ALSPAC*			
Adopted	2.4	—	
Spent time in care	1.2	—	
Poor family function	13.6	—	
*Cumulative psychosocial adversity score including additional items measured only in ALSPAC*			
0	32.4	—	
1	26.9	—	
2	15.8	—	
3+	24.9	—	

a Using a chi-squared test.

There was no evidence for associations of cumulative psychosocial adversity or childhood SEP with age acceleration in either cohort (Table 3). Results were similar for complete case analysis (Supplementary Material, Table S14) and for the extended cumulative psychosocial adversity score in ALSPAC (Supplementary Material, Table S15).

**Table 3.** Associations of childhood SEP and cumulative psychosocial adversity with methylation age acceleration

	ALSPAC age 29y	ALSPAC age 47y	NSHD age 53y
	(*n = *989)	(*n = *989)	(*n = *773)
Mean difference in methylation age acceleration (years) (95% CI)			
**Psychosocial adversity score**			
*Unadjusted*			
0 (ref)			
1	0.04 (−0.84, 0.93)	0.61 (−0.31, 1.54)	0.56 (−0.37, 1.48)
2	−0.84 (−1.89, 0.22)	0.41 (−0.70, 1.51)	−0.61 (−1.82, 0.61)
3	−0.27 (−1.20, 0.67)	0.03 (−0.96, 1.03)	−1.38 (−3.50, 0.74)
*Adjusted for childhood SEP*			
0 (ref)			
1	0.04 (−0.85, 0.92)	0.61 (−0.31, 1.53)	0.55 (−0.38, 1.48)
2	−0.84 (−1.90, 0.22)	0.40 (−0.70, 1.50)	−0.61 (−1.82, 0.61)
3	−0.25 (−1.19, 0.69)	0.05(−0.95, 1.05)	−1.39 (−3.51, 0.73)
*Additionally adjusted for adulthood SEP*			
0 (ref)			
1	0.03 (−0.85, 0.92)	0.59 (−0.34, 1.51)	0.58 (−0.35, 1.50)
2	−0.84 (−1.90, 0.22)	0.40 (−0.70, 1.50)	−0.54 (−1.75, 0.68)
3	−0.25 (−1.19, 0.69)	0.07 (−0.93, 0.61)	−1.30 (−3.42, 0.82)
**Childhood socioeconomic position (manual versus non-manual)**			
Unadjusted	−0.26 (−0.98, 0.46)	−0.31 (−1.08, 0.46)	0.13 (−0.69, 0.95)
Adjusted for cumulative psychosocial adversity	−0.26 (−0.98, 0.46)	−0.29 (−1.06, 0.48)	0.12 (−0.70, 0.94)
Additionally adjusted for adult SEP	−0.24 (−0.98, 0.50)	−0.18 (−0.97, 0.61)	0.30 (−0.54, 1.14)

Sexual abuse was strongly associated with age acceleration at both ALSPAC time points, which remained after adjustments for childhood and adulthood SEP, e.g. sexual abuse was associated with a 3.41 years higher DNA methylation age acceleration (95% CI 1.53 to 5.29) at the 47-year time point, after adjusting for both childhood and adulthood SEP. Sexual abuse associations were similar in complete case, non-imputed data (Supplementary Material, Table S17; Figs S1 and S2). Parental mental illness was associated with negative age acceleration in the ALSPAC 29-year methylation data, and the association remained with adjustment for childhood and adulthood socioeconomic status, but no evidence of association was seen in the older ALSPAC sample or in NSHD. There was no evidence of association between any of the other individual measures of psychosocial adversity and methylation age acceleration (Table 4 for unadjusted results, Supplementary Material, Table S16 for adjusted results). There was no evidence that the association between the cumulative psychosocial adversity score and methylation age acceleration differed according to adult SEP (Table 5).

**Table 4.** Associations between all forms of psychosocial adversity and methylation age acceleration (unadjusted)

	ALSPAC 29-year data	ALSPAC 47-year data	NSHD at 53 years
	(*n = *989)	(*n = *989)	(*n = *773)
Mean difference in methylation age acceleration (years) (95% CI)			
Parental physical illness	−0.37 (−1.11, 0.38)	0.11 (−0.68, 0.90)	−0.61 (−1.51, 0.29)
Parental absence	0.22 (−0.68, 1.13)	−0.04 (−0.99, 0.90)	0.76 (−1.90, 3.42)
Childhood physical illness	−0.71 (−2.27, 0.84)	0.09 (−1.52, 1.69)	0.07 (−1.09, 1.22)
Parental mental illness	−0.79 (−1.56, −0.03)	−0.03 (−0.79, 0.74)	2.41 (−0.59, 5.42)
Sub-optimal maternal bonding	−0.10 (−1.09, 0.88)	−0.13 (−1.10, 0.84)	−0.04 (−1.08, 1.01)
Parental divorce/separation	0.28 (−0.71, 1.27)	−0.56 (−1.60, 0.47)	−0.97 (−2.68, 0.74)
Parental death	0.72 (−0.87, 2.30)	0.29 (−1.33, 1.91)	−0.26 (−1.82, 1.30)
Child maltreatment	0.05 (−0.76, 0.86)	0.12 (−0.76, 1.01)	−1.26 (−2.93, 0.41)
Adoption	0.17 (−2.00, 2.34)	−0.85 (−3.13, 1.43)	n/a
Physical cruelty	0.84 (−1.04, 2.72)	0.15 (−1.81, 2.12)	n/a
Emotional cruelty	−0.28 (−1.52, 0.95)	−0.32 (−1.61, 0.96)	n/a
Physical neglect	−0.83 (−3.60, 1.95)	−2.06 (−5.40, 1.27)	n/a
Emotional neglect	0.28 (−0.57, 1.13)	−0.12 (−1.01, 0.77)	n/a
Spent time in care	−0.35 (−3.44, 2.75)	−1.43 (−4.72, 1.86)	n/a
Poor family function	0.91 (−0.13, 1.95)	−0.48 (−1.71, 0.74)	n/a
Sexual abuse	2.74 (0.93, 4.56)	3.34 (1.47, 5.22)	n/a

**Table 5.** Association between cumulative psychosocial adversity score and methylation age acceleration adjusted for childhood SEP, stratified by SEP in adulthood

	Mean difference in epigenetic age acceleration (years) (95% CI) comparing categories of the cumulative psychosocial adversity score		
	ALSPAC 29-year data	ALSPAC 47-year data	NSHD at 53 years
**Low adult SEP**	(*n =* 212)	(*n =* 197)	(*n =* 241)
0 (ref)			
1	−0.76 (−2.39, 0.88)	−0.65 (−2.74, 1.43)	0.09 (−1.56, 1.74)
2	−0.51 (−2.46, 1.43)	1.72 (−0.75, 4.20)	−0.75 (−2.94, 1.45)
3	−0.52 (−2.49, 1.45)	−0.72 (−3.20, 1.77)	−2.74 (−6.24, 0.76)
**High adult SEP**	(n= 432)	(n= 411)	(n= 397)
0 (ref)			
1	−0.08 (−1.29, 1.13)	0.56 (−0.63, 1.75)	1.20 (0.02, 2.39)
2	−1.18 (−2.68, 1.13)	−0.26 (−1.72, 1.20)	−0.09 (−1.77, 1.59)
3	−0.19 (−1.64, 1.27)	0.09 (−1.32, 1.50)	−0.50 (−3.48, 2.48)
**P for interaction**	0.74	0.31	0.17

## Discussion

In this study, sexual abuse was associated with DNA methylation age acceleration of approximately 3 years in analysis of data from the mothers of the ALSPAC study. This association was robust to adjustment for both childhood and adulthood SEP. We did not identify associations of low SEP, a cumulative score of total psychosocial adversity, or other individual types of psychosocial adversity with DNA methylation age acceleration. Parental mental illness was associated with negative DNA methylation age acceleration in one time point in the ALSPAC mothers, but this association did not replicate in the older time point in the ALSPAC mothers, nor in NSHD.

The association between sexual abuse and DNA methylation age acceleration is intriguing given the known associations between sexual abuse and a wide range of adverse outcomes (28), and the previously reported association between higher DNA methylation age and premature mortality (23). Unfortunately, sexual abuse was not measured in the NSHD cohort, so we were unable to replicate this finding to determine whether it is likely due to chance. A recent study of high risk inner-city youth (*n = *124, 68% of whom reported at least one form of maltreatment) identified differentially methylated probes for physical abuse (34 probes), sexual abuse (7 probes) and physical neglect (118 probes). No differentially methylated probes were identified for emotional abuse or neglect (29). Several other studies have also examined associations between child abuse and DNA methylation, but most use composite measures of abuse or maltreatment and are therefore unable to assess whether methylation changes differ for sexual versus physical or emotional abuse (15,30–33). This is important as, although there is co-occurrence between different types of adversity, we see here that not everyone who has experienced sexual abuse has also experienced other forms of adversity. In our analysis, a broader measure of child maltreatment and measures of physical and emotional cruelty were not associated with DNA methylation age. To our knowledge, no other studies have examined associations of sexual abuse or other forms of psychosocial adversity with DNA methylation age acceleration.

The reasons for lack of associations with methylation age acceleration for SEP and measures of psychosocial adversity other than sexual abuse are unclear. It is possible that our *a priori* hypothesis that adversity would manifest in higher age acceleration was incorrect, and that methylation as a biomarker of biological age does not reflect the influences of early life adversity, other than for sexual abuse (although this finding requires replication as discussed above). That said, it is possible that other changes to DNA methylation, not considered in this study, may be more relevant biological markers for psychosocial adversity, since associations between various forms of adversity and epigenetic changes at multiple sites on the genome have been identified in previous studies (16). Alternatively, adversity during specific developmental time periods could affect methylation age; only an accumulation of adversity from conception to age 17 years was measured here. Another possible explanation for our findings is that individuals who experience adverse childhood experiences but remain active participants of a cohort study (i.e. those people who are exposed to low SEP or psychosocial adversity but remain engaged in the cohorts) are more resilient than average and perhaps possess characteristics that protect them from the adverse consequences of their early life experiences. Associations may also only exist in the short-term and not persist (34) given that, in our study, there was a long time lag between the exposures (childhood) and the measures of methylation age acceleration (29, 47 and 53 years). This has also been documented in a previous study which shows non-persistence of methylation differences at some loci from birth to adolescence (34).

### Strengths and limitations

A key strength of this study is the use of two cohort studies with comparable data on childhood adversity. We considered a large number of types of psychosocial adversity, analysing them separately in case of distinct associations with methylation age acceleration, and jointly to account for their known co-occurrence and cumulative effects on long-term outcomes (24–27). We used the Horvath algorithm for estimating methylation age (17) and although other procedures are available(35), the Horvath method was validated using a range of biological samples, which is appropriate given we had blood samples in ALSPAC and buccal samples in NSHD (36).

We used a categorical cumulative score for all analyses as there was evidence of non-linearity for the 47-year ALSPAC sample (*P* = 0.03) using a likelihood ratio test comparing models including the score as a continuous or a categorical variable. We may not have had sufficient statistical power to detect small associations due to the sample size; post hoc power calculations suggest we had power to detect a difference of 1 year of age acceleration in ALSPAC and just over 1 year of age acceleration in NSHD. Furthermore, the prevalence of some types of adversity was low, which may have also reduced our power to detect associations. That said, the results were similar for the categorical cumulative score (which did not have low prevalence) and for ALSPAC and NSHD. It should be noted that due to the sample origin in NSHD, we could not adjust for cell count, as done so in ALSPAC. However, adjusting for cell counts in ALSPAC did not alter the results.

The summed score method of assessing cumulative psychosocial adversity assumes that each adverse experience has the same direction and magnitude of association with epigenetic age, which may be an unrealistic assumption (37). Other data-driven approaches are available that weight adverse exposures based the correlations between them (e.g. factor analysis), and this was our *a priori* preferred analysis strategy. However, model fit was very poor in NSHD, potentially due to the lower associations between the adversity measures in this cohort, precluding the use of these models. Reassuringly, however, analysis in ALSPAC demonstrated that results were unchanged when using the factor analysis or simple sum score approaches.

We observed a large mean difference between chronological and methylation age in NSHD; this may be due to the methylation data being derived from buccal samples in this cohort, but should not bias our results as the distribution of age acceleration should be unaffected. In contrast to the original study by Horvath et al used to develop the measure of epigenetic age (17), we found low Pearson's correlation coefficients between chronological age and predicted/epigenetic age. As demonstrated in other cohorts (22,23), this likely reflects the low variability of chronological age amongst our participants, as high correlations (such as *r =* 0.96 which was observed in the aforementioned study) have been observed in data sets comprised of subjects with a wide range of chronological ages. Adversity measures were self-reported in adulthood in the ALSPAC study, and in the NSHD study were primarily parent-reported during childhood. There is no gold standard method for measuring psychosocial adversity, and parent- and self-reports may lead to underestimation of differing types of adversity. It should also be noted that maltreatment was generated from multiple questions about different types of adverse experiences in ALSPAC. It is possible that pregnancy induces changes to epigenetic age that could have affected our results for the first time point in the ALSPAC mothers, however, it is reassuring that similar results were observed with all outcomes and, largely, between the two ALSPAC mothers' time points. Finally, our analysis is restricted to women, so our conclusions may not generalise to men.

## Conclusions

In conclusion, sexual abuse was associated with DNA methylation age acceleration by approximately 3 years. We found no evidence of associations of low SEP, a wide range of other types of psychosocial adversity or a cumulative score of psychosocial adversity in childhood with methylation age acceleration in adulthood. Further large, well-characterised studies with detailed measures of childhood adversity are needed to confirm our findings. Changes to DNA methylation age, as a marker of biological ageing, may potentially mediate associations between sexual abuse and adverse outcomes.

## Materials and Methods

### Data

The Avon Longitudinal Study of Parents and Children (ALSPAC) is a prospective, population-based birth cohort study that recruited 14 541 pregnant women residents in Avon, UK, with expected delivery dates between the 1 April 1991 and 31 December 1992 (38). The mothers, their partners and the index child have been followed-up via clinics, questionnaires and links to routine data. The mothers of this cohort form the participants for this study. The ALSPAC mothers in this study were mostly born in the early 1960s. The study website contains details of all the data that are available through a fully searchable data dictionary (http://www.bris.ac.uk/alspac/researchers/data-access/data-dictionary/; date last accessed February 2, 2018). The Accessible Resource for Integrated Epigenomic Studies (ARIES) project (39) includes 1018 mother-child pairs from ALSPAC that had DNA methylation measured using the Infinium HumanMethylation450BeadChip (Illumina, Inc). Details of how to access the data for this cohort are described elsewhere (39). Our analysis uses the mothers' DNA methylation data, which were generated from peripheral blood samples taken during pregnancy (mean age 29 years) and at a follow up clinic 17 years later (mean age 47 years). Ethical approval was obtained from the ALSPAC Ethics and Law Committee and the Local Research Ethics Committees.

The MRC National Survey of Health and Development (NSHD) is the UK's oldest birth cohort (40). It is based on a nationally representative sample of 5362 births out of all the single births that took place within marriage in one week in March 1946 in England, Scotland and Wales. DNA methylation was measured for a subsample of 790 women in the cohort using the Infinium HumanMethylation450BeadChip (Illumina, Inc) on buccal cell samples taken at age 53 years (41). All women gave written informed consent for their samples to be used in genetic studies of health, and the Central Manchester Ethics Committee approved the use of these samples for epigenetic studies of health in 2012. Details of how to access these data can be found online (http://www.nshd.mrc.ac.uk/data.aspx; date last accessed February 2, 2018); data requests should be submitted to mrclha.swiftinfo@ucl.ac.uk.

### Psychosocial adversity during childhood

The following types of psychosocial adversity were assessed in both ALSPAC and NSHD: maltreatment (neglect or abuse of any kind), sub-optimal maternal bonding, childhood physical illness, parental mental illness, absence of the mother or father in the household, parental physical illness or disability, parental divorce or separation and death of mother or father in childhood (see Supplementary Material for further details). In ALSPAC, women reported adverse childhood experiences retrospectively in questionnaires administered at the time of enrolment into the study (mean age 28 years), through pregnancy and postnatally. In NSHD, adverse childhood experiences were reported in interviews and questionnaires prospectively at age 4 years (or at 7 or 11 years if missing), except for parental bonding and maltreatment which were recalled when participants were age 43. The following additional measures were only available in ALSPAC: adoption, time spent in local authority care, and family functioning (i.e. the relationship between the mother and father).

### Socioeconomic position

Childhood SEP was assessed using father's occupational social class using the British Registrar General's Social Classification, and dichotomised as manual or non-manual. In ALSPAC this was self-reported by the women on entry to the cohort; in NSHD, it was reported by the mothers during health visitor interviews at age 4 years (or at 7 or 11 years if missing). For adult SEP, highest current occupational social class of the woman or their partner was used (reported in late pregnancy in ALSPAC and at age 53 years in NSHD). Adult SEP was dichotomised as manual or non-manual in NSHD. Due to the low prevalence of ‘manual' social class in the ALSPAC sample, adult SEP was coded as ‘high' (professional, managerial and technical occupations) or ‘low' (skilled [non-manual and manual], semi-skilled and unskilled occupations).

### Epigenetic age

In ALSPAC, women had methylation measured during pregnancy (mean age 29 years) and/or at a follow up clinic 17 years later (mean age 47 years). In NSHD, women had methylation measured at age 53 years. Following extraction, DNA was bisulfite converted using the Zymo EZ DNA MethylationTM kit (Zymo, Irvine, CA, USA). Infinium HumanMethylation450 BeadChips were used to measure genome-wide DNA methylation levels at over 485 000 CpG sites. The arrays were scanned using an Illumina iScan, with initial quality review using GenomeStudio. The level of methylation is expressed as a ‘beta' value (β-value), ranging from 0 (no cytosine methylation) to 1 (complete cytosine methylation). β-Values are reported as percentages. Several quality control steps were included in the laboratory pipeline, they are described in detail elsewhere (34). Epigenetic age for each available measure was derived by applying Horvath's epigenetic clock calculator (17) to raw β-values as previously described (36). Epigenetic age acceleration was defined as the residuals from a linear regression of epigenetic age on chronological age, adjusted for blood cell heterogeneity (ALSPAC only) by way of estimated cell-type proportions (42).

### Statistical analysis

In both cohorts, we restricted analysis to females who had data on at least one type of psychosocial adversity during childhood and epigenetic age in adulthood. For ALSPAC, participants had to have at least one of the two measures of epigenetic age, although time points were then analysed separately. All outcomes were analysed separately. Our *a priori* preferred analysis strategy for assessing cumulative psychosocial adversity was to create a data-driven weighted score using confirmatory factor analysis (since theory-driven approaches to weighting are highly subjective). However, this approach was not successful in the NSHD cohort; model fit was poor and factor loadings for all psychosocial adversity variables except for parental bonding variables (lack of care and overprotection) were low. Details of the model fit and factor loadings in the NSHD cohort are provided for information in the Supplementary Material. Thus, in order to maximise power and enable comparability of estimates from the two cohorts, we present results using a summary score of the eight binary measures of psychosocial adversity measured in both cohorts. This is a common approach used in the early life adversity literature (25), but has important limitations (37) in that it makes the unrealistic assumption that all of the separate types of psychosocial adversity have the same direction and magnitude of association with the outcome. That said, the results from the factor analysis in ALSPAC were similar to those using the summary score (full methods and results are presented in Supplementary Material, Tables S1–S9). Linearity of the association between the cumulative psychosocial adversity score and epigenetic age acceleration was assessed with a likelihood ratio test comparing models including the score as a continuous or a categorical variable.

To increase power and minimise selection bias, multiple imputation was used to impute data for participants who met the inclusion criteria. The imputation equations included measures of adversity; chronological age; DNA methylation age and age acceleration; childhood and adulthood SEP; fathers highest qualification; mothers highest qualification (NSHD only). The multivariate multiple imputation created 20 copies of the data in which missing values were imputed, with an appropriate level of randomness, by chained equations. The main analysis results are obtained by averaging across the results from each of these 20 datasets using Rubin's rules (43). This procedure appropriately modifies the standard errors for regression coefficients (used to calculate p-values and 95% confidence intervals) to take account of uncertainty in both the imputations and the estimate.

Multivariable linear regression models were used to assess the associations of childhood SEP, each separate type of psychosocial adversity, and the scores of cumulative psychosocial adversity with epigenetic age acceleration in the following models: (i) unadjusted (ii) mutually adjusted for childhood SEP and the cumulative psychosocial adversity score and (iii) additionally adjusted for potential mediation by adult SEP. We also assessed whether associations of childhood SEP and the cumulative psychosocial adversity score with epigenetic age acceleration differed according to adult SEP using likelihood ratio tests for interaction tests. This was intended to test the hypothesis that associations may only be present in people who are of low SEP in adulthood, which has also been shown to have health implications in later life (1,44,45). Sensitivity analyses were conducted in which analyses were restricted to participants with complete data on all variables, and using a second summary score for cumulative psychosocial adversity in ALSPAC including additional items only available in this cohort. Adversity scores were coded as 0, 1, 2, 3+.

## Supplementary Material

Supplementary Material is available at *HMG* online.

## Supplementary Material

Supplementary Data
