# Supplementary material for: Psychosocial adversity and socioeconomic position during childhood and epigenetic age: analysis of two prospective cohort studies
Source: Hum Mol Genet. 2018 Jan 22;27(7):1301–8. doi: 10.1093/hmg/ddy036 (PMC5985722; doi:10.1093/hmg/ddy036)
Supplement: Supplementary Data [file HMG_27_7_1301_s1.docx]

Supplementary material for:

**Psychosocial adversity and socioeconomic position during childhood and epigenetic age: analysis of two prospective cohort studies**

Rebecca B Lawn^1,2^*, Emma L Anderson^1,3^, Matthew Suderman^1,3^, Andrew J Simpkin^1,3^, Tom R Gaunt^1,3^, Andrew E Teschendorff ^5,6,7^, Martin Widschwendter ^5^, Rebecca Hardy^4^, Diana Kuh^4^, Caroline L Relton^1,3^, Laura D Howe^1,3^

*Corresponding author: rebecca.lawn@bristol.ac.uk. School of Experimental Psychology, University of Bristol, 12a Priory Road, Bristol BS8 1TU, UK. +44 (0)117 3310495

1 – MRC Integrative Epidemiology Unit at the University of Bristol, UK

2 – School of Experimental Psychology, University of Bristol, UK

3 – Department of Population Health Sciences, Bristol Medical School, University of Bristol, UK

4 – MRC Unit for Lifelong Health and Ageing at University College London, UK

5- Department of Women’s Cancer, University College London, 74 Huntley Street, WC1E 6AU, UK.

6- UCL Cancer Institute, University College London, 72 Huntley Street, WC1E 6BT, UK.

7 - CAS-Max-Planck Partner Institute for Computational Biology, Shanghai Institute for Biological Sciences, 320 Yueyang Road, Shanghai 200031, China

**Psychosocial adversity variable descriptions: ALSPAC**

Sub-optimal maternal bonding

We used the score by Parker and colleagues (1). This was dichotomised as “neglectful parenting” (low care and high protection) versus all other categories. For mothers, scores of 27.0 and 13.5 are used as cut offs for care and protection, respectively. This score was generated for participants with all adversity data and multiple imputation conducted.

Maltreatment

Maltreatment was generated from multiple questions about different types of adverse experiences relating to parental physical cruelty, emotional cruelty, sexual abuse, physical neglect and emotional neglect.

Childhood physical illness

Participants were asked whether they had a serious physical illness before age 17 years.

Parental mental illness

We used multiple questions related to mother and father’s depression and nerves, and alcohol use. We also included a general question of whether either parent was mentally ill before the participant was 17 years old.

Parental absence

Absence of the mother or father in the household for ages 0-5, 6-11, and 12-16 were combined into a binary variable of present or absent.

Parental physical illness or disability

Two questions asking if either parent were disabled or physically ill were combined into one binary variable.

Parental divorce or separation

Participants were asked if their parents were divorced or separated which was then used as a binary variable.

Death of mother or father in childhood

Participants were asked if their parents had died which was then used as a binary variable.

Adoption

Whether participants were adopted was asked in a single variable.

Time spent in local authority care

It was asked whether participants had spent time in local authority care before the age of 17 years.

Family functioning

Family functioning related to the relationship between participants mother and father. A score was derived by summing binary variables of whether participants’ parents’ relationship was violent, affectionate, quarrelsome, happy, frightening, friendly, respectful, and remote. A cut off of 4 was then used to create a binary variable for family functioning.

Sexual abuse

Multiple questions were asked about sexual experiences (including unwanted experiences) before age 16. Questions were asked in relation to the type of experience, who was involved in the experience, whether or not the participant wanted it to happen, how old they were when it first happened and how often it happened, and from responses to these questions a composite variable of sexual abuse was derived by the ALSPAC data team.

**Psychosocial adversity variable descriptions: NSHD**

Sub-optimal maternal bonding

Parental bonding was assessed retrospectively at age 43 years with the Parental Bonding Instrument (PBI) by Parker and colleagues (1). This was dichotomised as “neglectful parenting” (low care and high protection) versus all other categories. For mothers, scores of 27.0 and 13.5 are used as cut offs for care and protection, respectively. This score was generated for participants with all adversity data and multiple imputation conducted.

Maltreatment

At age 43 years, maltreatment was assessed retrospectively by asking participants ‘as a child do you feel you were mistreated by your parents in any way?’

Parental physical illness or disability and parental mental illness

During a health visitor interview mothers reported if they or their partner had a serious physical or psychiatric illness during the first 15 years of their child's life. During a health visitor interview at age 15, mothers reported if they or their partner suffered from any physical or nervous complaints.

Parental absence

When their child was aged 6, mothers were asked about the longest amount of time they had ever been separated from their children.

Parental divorce or separation

Experience of parental divorce before age 16 years was recorded.

Childhood physical illness

Childhood physical illness up to age 5, 11 and 15 were combined into a binary variable for childhood illness before 15 years old.

Death of mother or father in childhood

Experience of parental death before age 16 years was recorded.

**Details of confirmatory factor analyses of adversity variables**

Multiple questions were available that relate to several types of adversity in ALSPAC. Factor analysis was used to combine these questions. First order factor analyses were conducted using Mplus version 7.31(2) to estimate continuous latent constructs from responses to multiple questions about maltreatment (neglect or abuse of any kind), maternal bonding, childhood physical illness, and parental mental illness, absence of the mother or father in the household, parental physical illness or disability, parental divorce or separation and death of mother or father in childhood. Tables 1, 4 and 7 below provide details of included questions and prevalence for each adverse exposure for included and excluded participants for 28yr ALSPAC, 47yr ALSPAC and NSHD, respectively. Excluded participants are those that had missing data for the exposure, outcome, or adult socioeconomic position. Tables 2, 5, and 8 show model fit indices and factor loadings (with larger numbers representing a better loading) for 28yr ALSPAC, 47yr ALSPAC and NSHD, respectively. Any value above 0.4 was considered to be an acceptable factor loading. To assess the fit of each factor model we used root-mean-square error of approximations (RMSEA); comparative fit index (CFI); and Tucker-Lewis fit index (TFI).(3)

*Cumulative psychosocial adversity in childhood*
A second order factor analysis model was conducted to estimate a latent score of cumulative psychosocial adversity from all of the first order continuous latent constructs listed above plus observed binary variables derived from questionnaires. Adverse experiences that are more strongly correlated with other adverse experiences (and therefore experiences that more likely co-occur) are assigned higher factor loadings, and therefore contribute more to the factor score. Tables below details the factor loadings for each of the variables in the second order factor in both ALSPAC 28yr (table 3), 47yr (table 6) and NSHD (table 9). In ALSPAC, model fit was reasonable given the complexity of the model. However, model fit in NSHD was poor meaning we were unable to estimate associations of the latent cumulative psychosocial adversity score with DNA methylation age acceleration in this cohort.

In ALSPAC, structural equation models were used to simultaneously conduct the factor analyses described above and estimate associations of the latent cumulative psychosocial adversity score with DNA methylation age acceleration, using linear regression (table 8 below).

S1. Comparison of included (n=904) and excluded participants in 28yr ALSPAC data.

| **Adversity questions asked** | **Included participants (n=904)** | **Excluded participants**  ****** | |  |
| --- | --- | --- | --- | --- |
|  |  |  |  |  |
|  |  |  |  |  |
|  | **% Prevalence (N)** | **N with available data ***** | **% Prevalence (N)** | **P for difference*** |
| **First order factors** |  |  |  |  |
| **Lack of care** |  |  |  |  |
| Did your mother speak to you in a warm and friendly voice? (RS) | 17.47 (155) | 11 283 | 21.40 ( 2414) | 0.01 |
| Did your mother help you as much as you needed? (RS) | 17.57 (156) | 11 319 | 19.92 (2255) | 0.09 |
| Did your mother seem emotionally cold to you? | 4.62 (41) | 11 295 | 4.90 (554) | 0.71 |
| Did your mother appear to understand your problems and worries? (RS) | 48.59 (431) | 11 327 | 49.62 (5620) | 0.56 |
| Was your mother affectionate towards you? (RS) | 28.75 (255) | 11 322 | 30.02 (3399) | 0.43 |
| Did your mother make you feel you were not wanted? | 2.59 (23) | 11 328 | 4.02 (455) | 0.04 |
| Did your mother talk things over with you? (RS) | 56.50 (500) | 11 335 | 55.57 (6299) | 0.59 |
| Did your mother praise you? (RS) | 41.99 (372) | 11 303 | 48.40 (5471) | <0.01 |
| Did your mother enjoy talking things over with you? (RS) | 18.92 (164) | 11 128 | 19.64 (2185) | 0.61 |
| Did your mother frequently smile at you? (RS) | 10.36 (91) | 11 223 | 10.55 (1184) | 0.86 |
| Did your mother seem to understand what you needed or wanted? (RS) | 20.23 (176) | 11 108 | 22.34 (2481) | 0.15 |
| Did your mother make you feel better when you were upset? (RS) | 11.31 (99) | 11 166 | 13.92 (1554) | 0.03 |
| **Overprotection** |  |  |  |  |
| Did your mother allow you to do things you liked doing? (RS) | 40.47 (359) | 11 346 | 42.29 (4,798) | 0.29 |
| Did your mother try to control what you did? | 26.02 (230) | 11 318 | 25.32 (2866) | 0.65 |
| Did your mother let you decide things for yourself? (RS) | 49.77 (441) | 11 328 | 55.11 (6243) | <0.01 |
| Did your mother give you the freedom you wanted? (RS) | 61.89 (549) | 11 322 | 64.38 (7289) | 0.14 |
| Did your mother tend to baby you? | 15.58 (138) | 11 252 | 19.74 (2221) | <0.01 |
| Did you feel you could not look after yourself unless your mother was around? | 12.03 (106) | 11 213 | 15.90 (1783) | <0.01 |
| Did your mother let you go out as often as you wanted? (RS) | 56.80 (497) | 11 126 | 54.13 (6022) | 0.13 |
| Was your mother overprotective of you? | 19.47 (170) | 11 186 | 21.15 (2366) | 0.24 |
| Did your mother allow you to dress in any way you pleased? (RS) | 45.72 (401) | 11 161 | 42.15 (4704) | 0.04 |
| **Maladaptive family functioning** |  |  |  |  |
| Was your parent's behaviour stable and predictable to you as a child? (RS) | 11.30 (101) | 11 333 | 12.61 (1429) | 0.25 |
| Did your parents have serious arguments? | 25.67 (230) | 11 390 | 26.87 (3061) | 0.43 |
| Was your parent’s relationship violent? | 11.36 (90) | 8032 | 13.72 (1102) | 0.06 |
| Was your parent’s relationship affectionate? (RS) | 11.10 (89) | 8141 | 12.62 (1027) | 0.22 |
| Was your parent’s relationship quarrelsome? | 74.72 (606) | 8187 | 77.98 (6384) | 0.03 |
| Was your parent’s relationship happy? (RS) | 4.36 (35) | 8209 | 5.18 (425) | 0.32 |
| Was your parent’s relationship frightening? | 18.92 (151) | 8086 | 19.23 (1555) | 0.83 |
| Was your parent’s relationship friendly? (RS) | 2.98 (24) | 8142 | 3.76 (306) | 0.27 |
| Was your parent’s relationship respectful? (RS) | 6.38 (51) | 8115 | 9.14 (742) | 0.01 |
| Was your parent’s relationship remote or distant? | 40.53 (323) | 8074 | 42.35 (3419) | 0.32 |
| **Parental mental illness** |  |  |  |  |
| Was either parent mentally ill before age 17 years? | 5.36 (48) | 11 390 | 4.12 (469) | 0.08 |
| Did your mother have depression or nerves? | 19.10 (165) | 10 841 | 22.14 (2400) | 0.04 |
| Did your mother have an alcohol problem? | 2.15 (19) | 11 207 | 2.76 (309) | 0.29 |
| Did your father have an alcohol problem? | 6.32 (55) | 10 912 | 6.46 (705) | 0.87 |
| Did your father have depression or nerves? | 8.58 (74) | 10 743 | 8.10 (870) | 0.62 |
| **Parental absence** |  |  |  |  |
| Was your mother in the household 6-11yrs? (RS) | 3.34 (30) | 11 545 | 7.81 (902) | <0.01 |
| Was your mother in the household 12-15yrs? (RS) | 3.23 (29) | 11 545 | 7.50 (866) | <0.01 |
| Was your father in the household 0-5yrs? (RS) | 4.00 (36) | 11 545 | 8.63 (996) | <0.01 |
| Was your father in the household 6-11yrs? (RS) | 8.23 (74) | 11 545 | 14.77 (1705) | <0.01 |
| Was your father in the household 12-16yrs? (RS) | 11.68 (105) | 11 545 | 19.21 (2218) | <0.01 |
| **Second order factor - total psychosocial adversity score** |  |  |  |  |
| **Continuous latent constructs of first order factor scores** |  |  |  |  |
| Lack of care factor | - | - | - | - |
| Overprotection factor | - | - | - | - |
| Maladaptive family functioning factor | - | - | - | - |
| Parental mental illness factor | - | - | - | - |
| Absence of mother or father in household factor | - | - | - | - |
| **Observed binary variables** |  |  |  |  |
| **Parental disability** |  |  |  |  |
| Was your mother or father disabled in any way? | 14.50 (128) | 11 230 | 12.41 (1394) | 0.07 |
| **Parent physical illness** |  |  |  |  |
| Before age 17 was either parent seriously ill? | 18.86 (169) | 11 390 | 17.71 (2017) | 0.39 |
| **Physical abuse** |  |  |  |  |
| Before age 17 was either parent physically cruel to you? | 3.46 (31) | 11 390 | 3.42 (390) | 0.96 |
| **Emotional abuse** |  |  |  |  |
| Before age 17 was either parent physically cruel to you? | 8.48 (76) | 11 390 | 7.57 (862) | 0.32 |
| **Emotional neglect** |  |  |  |  |
| Were you emotionally neglected as a child? | 19.41 (164) | 8734 | 22.12 (1932) | 0.07 |
| **Parental divorce or separation** |  |  |  |  |
| Before age 17 years were your parents separated or divorced? | 12.72 (114) | 11 390 | 17.40 (1982) | <0.01 |
| **Death of mother or father** |  |  |  |  |
| Before age 17 years did your mother or father die? | 4.69 (42) | 11 390 | 6.02 (686) | 0.10 |
| **Adopted or spent time in care (derived from both questions)** |  |  |  |  |
| Before age 17 years were you adopted? |  |  |  |  |
| Before age 17 years did you spend time in local authority care? | 3.01 (27) | 11 469 | 5.40 (619) | <0.01 |
| **Sexual abuse** |  |  |  |  |
| Unwanted or abusive intercourse | 29.38 (248) | 10 255 | 29.25 (3000) | 0.94 |
| **Child physical illness** |  |  |  |  |
| Did you have a serious physical illness before age 17 years? | 5.02 (45) | 11 390 | 4.78 (545) | 0.75 |

RS=Reverse Score. All prevalence estimates are given for the ‘Yes’ category, except when RS is indicated, where prevalence is given for the ‘No’ category.

* The difference between those included and excluded from the analysis was tested using a Pearson's chi-squared test

** Excluded participants are those that had missing data for the exposure, outcome, or adult socioeconomic position

*** The N with available data for the excluded participant's column relates to the number of excluded participants that had data for each of the variables included in our analysis

S 2. Model fit for the first order psychosocial adversity factors in 28yr ALSPAC data.

| **Adversity questions asked** | **Factor loadings** | **Model fit statistics** |
| --- | --- | --- |
| **Lack of care** |  |  |
| Did your mother speak to you in a warm and friendly voice? (RS) | 0.91 | Chi2 = <0.001 |
| Did your mother help you as much as you needed? (RS) | 0.91 | RMSEA = 0.048 |
| Did your mother seem emotionally cold to you? | 0.80 | CFI = 0.989 |
| Did your mother appear to understand your problems and worries? (RS) | 0.83 | TLI = 0.987 |
| Was your mother affectionate towards you? (RS) | 0.87 |  |
| Did your mother make you feel you were not wanted? | 0.67 |  |
| Did your mother talk things over with you? (RS) | 0.88 |  |
| Did your mother praise you? (RS) | 0.83 |  |
| Did your mother enjoy talking things over with you? (RS) | 0.89 |  |
| Did your mother frequently smile at you? (RS) | 0.90 |  |
| Did your mother seem to understand what you needed or wanted? (RS) | 0.91 |  |
| Did your mother make you feel better when you were upset? (RS) | 0.90 |  |
| **Overprotection** |  |  |
| Did your mother allow you to do things you liked doing? (RS) | 0.58 | Chi2 = <0.001 |
| Did your mother try to control what you did? | 0.60 | RMSEA = 0.100 |
| Did your mother let you decide things for yourself? (RS) | 0.74 | CFI = 0.861 |
| Did your mother give you the freedom you wanted? (RS) | 0.84 | TLI = 0.821 |
| Did your mother tend to baby you? | 0.58 |  |
| Did you feel you could not look after yourself unless your mother was around? | 0.68 |  |
| Did your mother let you go out as often as you wanted? (RS) | 0.70 |  |
| Was your mother overprotective of you? | 0.68 |  |
| Did your mother allow you to dress in any way you pleased? (RS) | 0.61 |  |
| **Maladaptive family functioning** |  |  |
| Was your parent's behaviour stable and predictable to you as a child? (RS) | 0.75 | Chi2 = <0.001 |
| Did your parents have serious arguments? | 0.85 | RMSEA = 0.051 |
| Was your parent’s relationship violent? | 0.92 | CFI = 0.973 |
| Was your parent’s relationship affectionate? (RS) | 0.81 | TLI = 0.967 |
| Was your parent’s relationship quarrelsome? | 0.61 |  |
| Was your parent’s relationship happy? (RS) | 0.90 |  |
| Was your parent’s relationship frightening? | 0.91 |  |
| Was your parent’s relationship friendly? (RS) | 0.92 |  |
| Was your parent’s relationship respectful? (RS) | 0.90 |  |
| Was your parent’s relationship remote or distant? | 0.76 |  |
| **Parental mental illness** |  |  |
| Was either parent mentally ill before age 17 years? | 0.87 | Chi2 = <0.01 |
| Did your mother have depression or nerves? | 0.72 | RMSEA = 0.045 |
| Did your mother have an alcohol problem? | 0.65 | CFI = 0.915 |
| Did your father have an alcohol problem? | 0.41 | TLI = 0.858 |
| Did your father have depression or nerves? | 0.35 |  |
| **Parental absence** |  |  |
| Was your mother in the household 6-11yrs? (RS) | 0.92 | Chi2=<0.001 |
| Was your mother in the household 12-15yrs? (RS) | 0.85 | RMSEA = 0.076 |
| Was your father in the household 0-5yrs? (RS) | 0.90 | CFI = 0.984 |
| Was your father in the household 6-11yrs? (RS) | 1.03 | TLI = 0.973 |
| Was your father in the household 12-16yrs? (RS) | 0.85 |  |

S 3. Model fit for the second order psychosocial adversity factors in 28yr ALSPAC data

| **Adversity questions asked** | **Factor loadings** | **Model fit statistics** |
| --- | --- | --- |
| Second order factor - total psychosocial adversity score |  |  |
| **Continuous first order factor scores** |  |  |
| Lack of care factor |  | Chi2 = <0.001 |
| Overprotection factor |  | RMSEA = 0.039 |
| Maladaptive family functioning factor |  | CFI = 0.907 |
| Parental mental illness factor |  | TLI = 0.903 |
| Absence of mother or father in household factor |  |  |
| **Observed binary variables** |  |  |
| **Parental disability** |  |  |
| Was your mother or father disabled in any way? | 1.00 |  |
| **Parent physical illness** |  |  |
| Before age 17 was either parent seriously ill? | 0.11 |  |
| **Physical abuse** |  |  |
| Before age 17 was either parent physically cruel to you? | 0.82 |  |
| **Emotional abuse** |  |  |
| Before age 17 was either parent physically cruel to you? | 0.9 |  |
| **Emotional neglect** |  |  |
| Were you emotionally neglected as a child? | 0.89 |  |
| **Parental divorce or separation** |  |  |
| Before age 17 years were your parents separated or divorced? | 0.72 |  |
| **Death of mother or father** |  |  |
| Before age 17 years did your mother or father die? | 0.15 |  |
| **Adopted or spent time in care (derived from both questions)** |  |  |
| Before age 17 years were you adopted? |  |  |
| Before age 17 years did you spend time in local authority care? | 0.31 |  |
| **Sexual abuse** |  |  |
| Unwanted or abusive intercourse | 0.34 |  |
| **Child physical illness** |  |  |
| Did you have a serious physical illness before age 17 years? | 0.23 |  |

S 4. Comparison of included (n=868) and excluded participants in 47yr ALSPAC data.

| **Adversity questions asked** | **Included participants (n=868)** | **Excluded participants**** | |  |
| --- | --- | --- | --- | --- |
|  | **% Prevalence (N)*** | **N with available data ***** | **% Prevalence (N)*** | **P for difference** |
| **First order factors** |  |  |  |  |
| **Lack of care** |  |  |  |  |
| Did your mother speak to you in a warm and friendly voice? (RS) | 17.74 (151) | 11 319 | 21.36 (2418) | 0.01 |
| Did your mother help you as much as you needed? (RS) | 17.98 (153) | 11 356 | 19.88 (2258) | 0.18 |
| Did your mother seem emotionally cold to you? | 4.48 (38) | 11 333 | 4.91 (557) | 0.57 |
| Did your mother appear to understand your problems and worries? (RS) | 47.94 (407) | 11 365 | 49.66 (5644) | 0.33 |
| Was your mother affectionate towards you? (RS) | 28.12 (239) | 11 359 | 30.06 (3415) | 0.23 |
| Did your mother make you feel you were not wanted? | 2.71 (23) | 11 366 | 4.00 (455) | 0.06 |
| Did your mother talk things over with you? (RS) | 55.78 (473) | 11 372 | 55.63 (6326) | 0.93 |
| Did your mother praise you? (RS) | 41.39 (351) | 11 341 | 48.43 (5492) | <0.01 |
| Did your mother enjoy talking things over with you? (RS) | 18.53 (154) | 11 164 | 19.66 (2195) | 0.43 |
| Did your mother frequently smile at you? (RS) | 10.23 (86) | 11 260 | 10.56 (1189) | 0.76 |
| Did your mother seem to understand what you needed or wanted? (RS) | 20.84 (174) | 11 143 | 22.28 (2483) | 0.33 |
| Did your mother make you feel better when you were upset? (RS) | 11.22 (94) | 11 203 | 13.92 (1559) | 0.03 |
| **Overprotection** |  |  |  |  |
| Did your mother allow you to do things you liked doing? (RS) | 40.12 (341) | 11 383 | 42.31 (4816) | 0.21 |
| Did your mother try to control what you did? | 26.12 (221) | 11 356 | 25.32 (2875) | 0.6 |
| Did your mother let you decide things for yourself? (RS) | 48.71 (414) | 11 364 | 55.17 (6270) | <0.01 |
| Did your mother give you the freedom you wanted? (RS) | 62.35 (530) | 11 359 | 64.34 (7308) | 0.25 |
| Did your mother tend to baby you? | 14.84 (126) | 11 289 | 19.78 (2233) | <0.01 |
| Did you feel you could not look after yourself unless your mother was around? | 11.98 (101) | 11 251 | 15.89 (1788) | <0.01 |
| Did your mother let you go out as often as you wanted? (RS) | 56.63 (474) | 11 164 | 54.15 (6045) | 0.16 |
| Was your mother overprotective of you? | 18.80 (157) | 11 224 | 21.20 (2379) | 0.10 |
| Did your mother allow you to dress in any way you pleased? (RS) | 46.67 (392) | 11 198 | 42.09 (4713) | 0.01 |
| **Maladaptive family functioning** |  |  |  |  |
| Was your parent's behaviour stable and predictable to you as a child? (RS) | 11.70 (100) | 11 372 | 12.57 (1430) | 0.45 |
| Did your parents have serious arguments? | 25.73 (221) | 11 427 | 26.87 (3070) | 0.47 |
| Was your parent’s relationship violent? | 11.20 (85) | 8065 | 13.73 (1107) | 0.05 |
| Was your parent’s relationship affectionate? (RS) | 10.64 (82) | 8172 | 12.65 (1034) | 0.11 |
| Was your parent’s relationship quarrelsome? | 73.94 (576) | 8219 | 78.04 (6414) | 0.01 |
| Was your parent’s relationship happy? (RS) | 4.15 (32) | 8240 | 5.19 (428) | 0.21 |
| Was your parent’s relationship frightening? | 18.82 (144) | 8119 | 19.24 (1562) | 0.78 |
| Was your parent’s relationship friendly? (RS) | 2.72 (21) | 8174 | 3.78 (309) | 0.13 |
| Was your parent’s relationship respectful? (RS) | 6.38 (49) | 8146 | 9.13 (744) | 0.01 |
| Was your parent’s relationship remote or distant? | 39.69 (304) | 8105 | 42.42 (3438) | 0.14 |
| **Parental mental illness** |  |  |  |  |
| Was either parent mentally ill before age 17 years? | 5.59 (48) | 11 427 | 4.10 (469) | 0.04 |
| Did your mother have depression or nerves? | 18.86 (156) | 10 878 | 22.15 (2409) | 0.03 |
| Did your mother have an alcohol problem? | 2.37 (20) | 11 244 | 2.74 (308) | 0.52 |
| Did your father have an alcohol problem? | 6.14 (51) | 10 952 | 6.47 (709) | 0.71 |
| Did your father have depression or nerves? | 8.85 (73) | 10 780 | 8.08 (871) | 0.44 |
| **Parental absence** |  |  |  |  |
| Was your mother in the household 6-11yrs? (RS) | 3.26 (28) | 11 584 | 7.80 (904) | <0.01 |
| Was your mother in the household 12-15yrs? (RS) | 3.14 (27) | 11 584 | 7.49 (868) | <0.01 |
| Was your father in the household 0-5yrs? (RS) | 4.19 (36) | 11 584 | 8.60 (996) | <0.01 |
| Was your father in the household 6-11yrs? (RS) | 8.49 (73) | 11 584 | 14.73 (1706) | <0.01 |
| Was your father in the household 12-16yrs? (RS) | 12.21 (105) | 11 584 | 19.15 (2218) | <0.01 |
| **Second order factor - total psychosocial adversity score** |  |  |  |  |
| **Continuous latent constructs of first order factor scores** |  |  |  |  |
| Lack of care factor | - |  | - | - |
| Overprotection factor | - |  | - | - |
| Maladaptive family functioning factor | - |  | - | - |
| Parental mental illness factor | - |  | - | - |
| Absence of mother or father in household factor | - |  | - | - |
| **Observed binary variables** |  |  |  |  |
| **Parental disability** |  |  |  |  |
| Was your mother or father disabled in any way? | 15.18 (128) | 11 270 | 12.37 (1394) | 0.02 |
| **Parent physical illness** |  |  |  |  |
| Before age 17 was either parent seriously ill? | 18.74 (161) | 11 427 | 17.72 (2025) | 0.45 |
| **Physical abuse** |  |  |  |  |
| Before age 17 was either parent physically cruel to you? | 3.38 (29) | 11 427 | 3.43 (392) | 0.93 |
| **Emotional abuse** |  |  |  |  |
| Before age 17 was either parent physically cruel to you? | 8.03 (69) | 11 427 | 7.60 (869) | 0.65 |
| **Emotional neglect** |  |  |  |  |
| Were you emotionally neglected as a child? | 19.09 (155) | 8767 | 22.14 (1941) | 0.04 |
| **Parental divorce or separation** |  |  |  |  |
| Before age 17 years were your parents separated or divorced? | 12.46 (107) | 11 427 | 17.41 (1989) | <0.01 |
| **Death of mother or father** |  |  |  |  |
| Before age 17 years did your mother or father die? | 4.77 (41) | 11 427 | 6.01 (687) | 0.14 |
| **Adopted or spent time in care** |  |  |  |  |
| Before age 17 years were you adopted? |  |  |  |  |
| Before age 17 years did you spend time in local authority care? | 3.03 (26) | 11 508 | 5.39 (620) | <0.01 |
| **Sexual abuse** |  |  |  |  |
| Unwanted or abusive intercourse | 29.84 (242) | 10 288 | 29.22 (3006) | 0.71 |
| **Child physical illness** |  |  |  |  |
| Did you have a serious physical illness before age 17 years? | 5.47 (47) | 11 427 | 4.75 (543) | 0.34 |

RS=Reverse Score. All prevalence estimates are given for the ‘Yes’ category, except when RS is indicated, where prevalence is given for the ‘No’ category.

* The difference between those included and excluded from the analysis was tested using a Pearson's chi-squared test

** Excluded participants are those that had missing data for the exposure, outcome, or adult socioeconomic position

*** The N with available data for the excluded participant's column relates to the number of excluded participants that had data for each of the variables included in our analysis

S 5. Model fit for the first order psychosocial adversity factors in 47yr ALSPAC data

| **Adversity questions asked** | **Factor loadings** | **Model fit statistics** |
| --- | --- | --- |
| **Lack of care** |  |  |
| Did your mother speak to you in a warm and friendly voice? (RS) | 0.93 | Chi2 = <0.001 |
| Did your mother help you as much as you needed? (RS) | 0.91 | RMSEA = 0.054 |
| Did your mother seem emotionally cold to you? | 0.79 | CFI = 0.986 |
| Did your mother appear to understand your problems and worries? (RS) | 0.82 | TLI = 0.983 |
| Was your mother affectionate towards you? (RS) | 0.85 |  |
| Did your mother make you feel you were not wanted? | 0.69 |  |
| Did your mother talk things over with you? (RS) | 0.87 |  |
| Did your mother praise you? (RS) | 0.83 |  |
| Did your mother enjoy talking things over with you? (RS) | 0.88 |  |
| Did your mother frequently smile at you? (RS) | 0.89 |  |
| Did your mother seem to understand what you needed or wanted? (RS) | 0.91 |  |
| Did your mother make you feel better when you were upset? (RS) | 0.90 |  |
| **Overprotection** |  |  |
| Did your mother allow you to do things you liked doing? (RS) | 0.60 | Chi2 = <0.001 |
| Did your mother try to control what you did? | 0.63 | RMSEA = 0.095 |
| Did your mother let you decide things for yourself? (RS) | 0.75 | CFI = 0.877 |
| Did your mother give you the freedom you wanted? (RS) | 0.82 | TLI = 0.842 |
| Did your mother tend to baby you? | 0.59 |  |
| Did you feel you could not look after yourself unless your mother was around? | 0.69 |  |
| Did your mother let you go out as often as you wanted? (RS) | 0.67 |  |
| Was your mother overprotective of you? | 0.70 |  |
| Did your mother allow you to dress in any way you pleased? (RS) | 0.60 |  |
| **Maladaptive family functioning** |  |  |
| Was your parent's behaviour stable and predictable to you as a child? (RS) | 0.77 | Chi2 = <0.005 |
| Did your parents have serious arguments? | 0.85 | RMSEA = 0.046 |
| Was your parent’s relationship violent? | 0.92 | CFI = 0.978 |
| Was your parent’s relationship affectionate? (RS) | 0.80 | TLI = 0.974 |
| Was your parent’s relationship quarrelsome? | 0.60 |  |
| Was your parent’s relationship happy? (RS) | 0.89 |  |
| Was your parent’s relationship frightening? | 0.91 |  |
| Was your parent’s relationship friendly? (RS) | 0.92 |  |
| Was your parent’s relationship respectful? (RS) | 0.91 |  |
| Was your parent’s relationship remote or distant? | 0.74 |  |
| **Parental mental illness** |  |  |
| Was either parent mentally ill before age 17 years? | 0.89 | Chi2 = <0.01 |
| Did your mother have depression or nerves? | 0.70 | RMSEA = 0.041 |
| Did your mother have an alcohol problem? | 0.75 | CFI = 0.939 |
| Did your father have an alcohol problem? | 0.41 | TLI = 0.898 |
| Did your father have depression or nerves? | 0.29 |  |
| **Parental absence** |  |  |
| Was your mother in the household 6-11yrs? (RS) | 0.91 | Chi2 = <0.001 |
| Was your mother in the household 12-15yrs? (RS) | 0.85 | RMSEA = 0.080 |
| Was your father in the household 0-5yrs? (RS) | 0.89 | CFI = 0.983 |
| Was your father in the household 6-11yrs? (RS) | 1.04 | TLI = 0.971 |
| Was your father in the household 12-16yrs? (RS) | 0.85 |  |

S 6. Model fit for the second order psychosocial adversity factors in 47yr ALSPAC data

| **Adversity questions asked** | **Factor loadings** | **Model fit statistics** |
| --- | --- | --- |
| **Second order factor - total psychosocial adversity score** |  |  |
| **Continuous latent constructs of first order factor scores** |  |  |
| Lack of care factor |  | Chi2 = <0.001 |
| Overprotection factor |  | RMSEA = 0.040 |
| Maladaptive family functioning factor |  | CFI = 0.903 |
| Parental mental illness factor |  | TLI = 0.898 |
| Absence of mother or father in household factor |  |  |
| **Observed binary variables** |  |  |
| **Parental disability** |  |  |
| Was your mother or father disabled in any way? | 1.00 |  |
| **Parent physical illness** |  |  |
| Before age 17 was either parent seriously ill? | 0.13 |  |
| **Physical abuse** |  |  |
| Before age 17 was either parent physically cruel to you? | 0.79 |  |
| **Emotional abuse** |  |  |
| Before age 17 was either parent physically cruel to you? | 0.89 |  |
| **Emotional neglect** |  |  |
| Were you emotionally neglected as a child? | 0.89 |  |
| **Parental divorce or separation** |  |  |
| Before age 17 years were your parents separated or divorced? | 0.73 |  |
| **Death of mother or father** |  |  |
| Before age 17 years did your mother or father die? | 0.18 |  |
| **Adopted or spent time in care** |  |  |
| Before age 17 years were you adopted? |  |  |
| Before age 17 years did you spend time in local authority care? | 0.26 |  |
| **Sexual abuse** |  |  |
| Unwanted or abusive intercourse | 0.37 |  |
| **Child physical illness** |  |  |
| Did you have a serious physical illness before age 17 years? | 0.21 |  |

S 7. Comparison of included (n=773) and excluded participants in NSHD data.

| **Adversity measures** | **Included participants (773)** | **Excluded participants**  ****** | |  |
| --- | --- | --- | --- | --- |
|  |  |  |  |  |
|  |  |  |  |  |
|  | **% Prevalence (N)** | **N with available data ***** | **% Prevalence (N)** | **P for difference*** |
| **First order factors** |  |  |  |  |
| **Lack of care** |  |  |  |  |
| Was your mother affectionate towards you? (RS) | 14.27 (102) | 2391 | 12.51 (299) | 0.22 |
| Did your mother make you feel better when you were upset? (RS) | 16.32 (118) | 2393 | 15.67 (375) | 0.68 |
| Did your mother enjoy talking things over with you? (RS) | 28.08 (203) | 2386 | 31.39 (749) | 0.90 |
| Did your mother want you to grow up? | 28.67 (203) | 2341 | 24.73 (579) | 0.04 |
| Did your mother help you as much as you needed? (RS) | 7.52 (54) | 2401 | 8.16 (196) | 0.58 |
| Did your mother seem to understand what you needed or wanted? (RS) | 27.36 (197) | 2378 | 25.82 (614) | 0.41 |
| Did your mother praise you? (RS) | 21.85 (158) | 2400 | 20.08 (482) | 0.30 |
| Did your mother make you feel you were not wanted? | 16.95 (119) | 2379 | 14.38 (342) | 0.09 |
| Did your mother frequently smile at you? (RS) | 15.55 (111) | 2384 | 16.23 (387) | 0.66 |
| Did your mother speak to you in a warm and friendly voice? (RS) | 5.20 (38) | 2432 | 6.13 (149) | 0.35 |
| Did your mother talk things over with you? (RS) | 20.06 (144) | 2384 | 18.16 (433) | 0.25 |
| Did your mother appear to understand your problems and worries? (RS) | 20.83 (150) | 2402 | 18.48 (444) | 0.16 |
| **Overprotection** |  |  |  |  |
| Did your mother tend to baby you? | 23.19 (163) | 2376 | 29.59 (703) | 0.001 |
| Did your mother try to control what you did? | 36.07 (259) | 2383 | 33.53 (799) | 0.208 |
| Did your mother let you decide things for yourself? (RS) | 22.35 (160) | 2385 | 21.51 (513) | 0.63 |
| Did your mother like you to make your own decisions? (RS) | 17.81 (127) | 2386 | 18.44 (440) | 0.70 |
| Did your mother try to make you dependent on her? | 23.82 (171) | 2390 | 23.89 (571) | 0.97 |
| Did your mother give you the freedom you wanted? (RS) | 39.00 (282) | 2401 | 33.28 (799) | 0.01 |
| Did your mother allow you to dress in any way you pleased? (RS) | 33.15 (241) | 2411 | 34.18 (824) | 0.61 |
| Did your mother invade your privacy? | 28.33 (202) | 2362 | 23.88 (564) | 0.02 |
| Did your mother allow you to do things you liked doing? (RS) | 10.39 (75) | 2403 | 11.03 (265) | 0.63 |
| Was your mother overprotective of you? | 31.67 (228) | 2389 | 30.89 (738) | 0.69 |
| Did your mother let you go out as often as you wanted? (RS) | 43.19 (311) | 2394 | 34.00 (814) | <0.001 |
| Did you feel you could not look after yourself unless your mother was around? | 20.20 (142) | 2382 | 23.13 (551) | 0.1 |
| **Second order factor - total psychosocial adversity score** |  |  |  |  |
| **Continuous latent constructs of first order factor scores** |  |  |  |  |
| Lack of care factor | - | - | - | - |
| Overprotection factor | - | - | - | - |
| **Observed binary variables** |  |  |  |  |
| **Parental divorce** | 5.82 (45) | 4587 | 5.89 (270) | 0.94 |
| **Parental mental illness** | 1.81 (14) | 4589 | 2.16 (99) | 0.54 |
| **Parental absence** | 2.33 (18) | 4589 | 1.87 (86) | <0.001 |
| **Parent physical illness** | 27.17 (210) | 4589 | 19.50 (895) | 0.40 |
| **Parental death** | 7.12 (55) | 4584 | 7.64 (350) | 0.61 |

S 8. Model fit for the first order psychosocial adversity factors in NSHD data

| **First order factors** | | **Factor loadings** | | **Model fit statistics** | |
| --- | --- | --- | --- | --- | --- |
| **Lack of care** | |  | |  | |
| Was your mother affectionate towards you? (RS) | | 0.91 | | Chi2 = <0.001 | |
| Did your mother make you feel better when you were upset? (RS) | | 0.83 | | RMSEA = 0.05 | |
| Did your mother enjoy talking things over with you? (RS) | | 0.85 | | CFI = 0.99 | |
| Did your mother want you to grow up? | | 0.22 | | TLI = 0.99 | |
| Did your mother help you as much as you needed? (RS) | | 0.91 | |  | |
| Did your mother seem to understand what you needed or wanted? (RS) | | 0.90 | |  | |
| Did your mother praise you? (RS) | | 0.84 | |  | |
| Did your mother make you feel you were not wanted? | | 0.41 | |  | |
| Did your mother frequently smile at you? (RS) | | 0.78 | |  | |
| Did your mother speak to you in a warm and friendly voice? (RS) | | 0.89 | |  | |
| Did your mother talk things over with you? (RS) | | 0.85 | |  | |
| Did your mother appear to understand your problems and worries? (RS) | | 0.92 | |  | |
| **Overprotection** | |  | |  | |
| Did your mother tend to baby you? | | 0.46 | | Chi2 = <0.001 | |
| Did your mother try to control what you did? | | 0.60 | | RMSEA = 0.15 | |
| Did your mother let you decide things for yourself? (RS) | | 0.78 | | CFI = 0.77 | |
| Did your mother like you to make your own decisions? (RS) | | 0.79 | | TLI = 0.72 | |
| Did your mother try to make you dependent on her? | | 0.56 | |  | |
| Did your mother give you the freedom you wanted? (RS) | | 0.85 | |  | |
| Did your mother allow you to dress in any way you pleased? (RS) | | 0.61 | |  | |
| Did your mother invade your privacy? | | 0.63 | |  | |
| Did your mother allow you to do things you liked doing? (RS) | | 0.66 | |  | |
| Was your mother overprotective of you? | | 0.58 | |  | |
| Did your mother let you go out as often as you wanted? (RS) | | 0.80 | |  | |
| Did you feel you could not look after yourself unless your mother was around? | | 0.68 | |  | |
| **Adversity measures** | **Factor loadings** | | **Model fit statistics** | |  |
| Second order factor - total psychosocial adversity score |  | |  | |  |
| **Continuous first order factor scores** |  | |  | |  |
| Lack of care factor | -0.73 | | Chi2 = <0.001 | |  |
| Overprotection factor | -0.90 | | RMSEA = 2.49 | |  |
| **Observed binary variables** |  | | CFI = 0.841 | |  |
| Parental divorce | 1.00 | | TLI = 0.828 | |  |
| Parental mental illness | -0.03 | |  | |  |
| Parental absence | 0.08 | |  | |  |
| Parent physical illness | -0.02 | |  | |  |
| Parental death | 0.003 | |  | |  |

S 9. Unadjusted associations between factor analysis of psychosocial adversity and DNA methylation age acceleration measured at 28yr and 47yr in ALSPAC.

|  | | 28yr | 47yr |
| --- | --- | --- | --- |
|  |  | (n = 904) | (n= 868) |
|  |  | Mean difference (95% CI) per SD higher psychosocial adversity | |
| **First Order Factors** | |  |  |
|  | Parental absence | 0.27 (-0.26, 0.80) | -0.01 (-0.61, 0.59) |
|  | Parental mental illness | -0.50 (-1.05, 0.05) | -0.05 (-0.67, 0.57) |
|  | Overprotection | 0.05 (-0.34, 0.44) | -0.13 (-0.54, 0.28) |
|  | Lack of care | -0.30 (-0.68, 0.08) | -0.38 (-0.79, 0.02) |
|  | Maladaptive family functioning | 0.26 (-0.17, 0.69) | -0.38 (-0.85, 0.09) |
| **Observed variables** | |  |  |
|  | Parental disability | -0.10 (-1.07, 0.87) | -0.01 (-1.03, 1.00) |
|  | Parent physical illness | -0.09 (-0.95, 0.77) | 0.22 (-0.70, 1.14) |
|  | Physical abuse | 0.47 (-1.38, 2.31) | -0.09 (-2.08, 1.90) |
|  | Emotional abuse | -0.43 (-1.64, 0.79) | -0.46 (-1.78, 0.86) |
|  | Parental divorce or separation | 0.36 (-0.66, 1.37) | -0.51 (-1.60, 0.58) |
|  | Death of mother or father | 0.87 (-0.73, 2.47) | 0.14 (-1.54, 1.83) |
|  | Emotional neglect | 0.09 (-0.78, 0.96) | -0.32 (-1.25, 0.61) |
|  | Child physical illness | -0.61 (-2.16, 0.93) | 0.10 (-1.48, 1.68) |
|  | Adopted or spent time in care | -0.21 (-2.19, 1.77) | -0.82 (-2.92. 1.28) |
|  | Sexual abuse | 0.38 (-0.38, 1.14) | 0.85 (0.04, 1.66) |
| **Total psychosocial adversity** | | -0.05 (-0.44, 0.34) | -0.35 (-0.74, 0.05) |

S 10. Number of participants with non-missing data and percentage of imputed data for ALSPAC and NSHD.

|  | **ALSPAC (imputed N = 989)** | | **NSHD (imputed N = 773)** | |
| --- | --- | --- | --- | --- |
|  | Number of eligible participants with non-missing data | % Imputed | Number of participants with non-missing data | % Imputed |
| SEP in childhood | 836 | 15.47 | 764 | 1.16 |
| SEP in adulthood | 954 | 3.54 | 773 | - |
| **Psychosocial adversity before 17 years:** |  | | |  |
| Parent physically ill | 972 | 1.72 | 773 | - |
| Parent absent | 978 | 1.11 | 773 | - |
| Child illness | 969 | 2.02 | 773 | - |
| Parent mentally ill | 920 | 6.98 | 773 | - |
| Sub-optimal maternal bonding | 888 | 10.21 | 675 | 12.68 |
| Parents separated | 969 | 2.02 | 773 | - |
| Parent died | 969 | 2.02 | 773 | - |
| Child maltreatment | 898 | 9.2 | 701 | 9.31 |
| *Items in maltreatment variable in ALSPAC:* | | | |  |
| Physical cruelty | 969 | 2.02 | - |  |
| Emotional cruelty | 969 | 2.02 | - |  |
| Physical neglect | 914 | 7.58 | - |  |
| Emotional neglect | 914 | 7.58 | - |  |
| Sexual abuse | 969 | 2.02 | - |  |
| *Additional items measured only in ALSPAC:* | | | |  |
| Adopted | 974 | 1.52 | - |  |
| Spent time in care | 938 | 5.16 | - |  |
| Poor family function | 833 | 15.77 | - |  |
| **Age variables:** |  |  |  |  |
| DNA methylation age |  |  | 773 | - |
| *28yr* | 937 | 5.26 |  |  |
| *47yr* | 956 | 3.34 |  |  |
| DNA methylation age acceleration |  |  | 773 | - |
| *28yr* | 937 | 5.26 |  |  |
| *47yr* | 896 | 9.40 |  |  |

S 11. Associations (%) between psychosocial adversity measures in ALSPAC complete case data

|  | **Child physical illness** | **Parental absence from household** | **Parental mental illness** | **Sub-optimal maternal bonding** | **Parental physical illness or disability** | **Parental divorce or separation** | **Parental death** | **Maltreatment** |
| --- | --- | --- | --- | --- | --- | --- | --- | --- |
| **Prevalence (%)** | 5.3 | 16.2 | 32.5 | 16.5 | 28.7 | 13.3 | 5.1 | 23.0 |
| ***Percentage of these who also experienced:*** | | | | | | | | |
| Child physical illness | - | 5.26 | 5.88 | 6.43 | 9.09 | 7.14 | 6.25 | 8.42 |
|  |  |  |  |  |  |  |  |  |
| Parental absence from household | 16.00 | - | 25.00 | 16.67 | 16.61 | 74.19 | 66.67 | 30.50 |
|  |  |  |  |  |  |  |  |  |
| Parental mental illness | 34.69 | 54.41 | - | 46.32 | 40.70 | 52.21 | 36.59 | 53.44 |
|  |  |  |  |  |  |  |  |  |
| Sub-optimal maternal bonding | 20.00 | 17.27 | 23.68 | - | 24.69 | 20.51 | 21.43 | 40.88 |
|  |  |  |  |  |  |  |  |  |
| Parental physical illness or disability | 49.02 | 30.26 | 36.21 | 41.84 | - | 22.22 | 45.83 | 38.12 |
|  |  |  |  |  |  |  |  |  |
| Parental divorce or separation | 17.65 | 60.53 | 20.42 | 17.14 | 10.18 | - | 16.67 | 26.73 |
|  |  |  |  |  |  |  |  |  |
| Parental death | 5.88 | 21.05 | 5.19 | 6.43 | 8.00 | 6.35 | - | 7.43 |
|  |  |  |  |  |  |  |  |  |
| Maltreatment | 36.17 | 44.20 | 37.55 | 57.36 | 29.62 | 45.76 | 32.61 | - |

Note: Percentages presented represent the % of participants with the adversity listed in the top row who also experienced each other type of adversity measures.

|  | **Child physical illness** | **Parental absence from household** | **Parental mental illness** | **Sub-optimal maternal bonding** | **Parental physical illness or disability** | **Parental divorce or separation** | **Parental death** | **Physical cruelty** | **Emotional cruelty** | **Physical neglect** | **Emotional neglect** | **Sexual abuse** |
| --- | --- | --- | --- | --- | --- | --- | --- | --- | --- | --- | --- | --- |
| **Prevalence (%)** | 5.3 | 16.2 | 32.5 | 16.5 | 28.7 | 13.3 | 5.1 | 3.5 | 8.4 | 1.5 | 20.2 | 3.8 |
| ***Percentage of these who also experienced:*** | | | | | | | | | | | | |
| Physical cruelty | 9.80 | 6.58 | 6.23 | 10.71 | 5.09 | 7.94 | 4.17 | - | 31.65 | 46.15 | 15.17 | 25.17 |
| Emotional cruelty | 19.61 | 18.42 | 15.92 | 26.43 | 15.27 | 23.02 | 10.42 | 75.76 | - | 76.92 | 35.39 | 28.57 |
| Physical neglect | 4.26 | 3.52 | 3.27 | 5.26 | 2.67 | 2.54 | 2.17 | 18.75 | 13.33 | - | 6.49 | 6.06 |
| Emotional neglect | 29.79 | 39.16 | 33.70 | 54.89 | 26.34 | 39.83 | 28.26 | 84.38 | 84.00 | 92.31 | - | 78.79 |
| Sexual abuse | 3.92 | 7.89 | 6.92 | 7.14 | 4.00 | 6.35 | 4.17 | 27.27 | 12.66 | 15.38 | 14.61 | - |

S 12. Associations (%) between individual neglect items forming the maltreatment variable in ALSPAC and psychosocial adversity measures in complete case data

Note: Percentages presented represent the % of participants with the adversity listed in the top row who also experienced each other type of adversity measures.

S 13. Associations (%) between psychosocial adversity measures in NSHD complete case data

|  | **Child physical illness** | **Parental absence from household** | **Parental mental illness** | **Sub-optimal maternal bonding** | **Parental physical illness or disability** | **Parental divorce or separation** | **Parental death** | **Maltreatment** |
| --- | --- | --- | --- | --- | --- | --- | --- | --- |
| **Prevalence (%)** | 14.1 | 2.3 | 1.8 | 20.7 | 27.2 | 5.8 | 7.1 | 7.2 |
| ***Percentage of these who also experienced:*** | | | | | | | | |
| **Child physical illness** | - | 11.11 | 14.29 | 12.32 | 15.24 | 13.33 | 10.91 | 12.24 |
|  |  |  |  |  |  |  |  |  |
| **Parental absence from household** | 1.83 | - | 0.00 | 0.72 | 1.90 | 6.67 | 18.18 | 2.04 |
|  |  |  |  |  |  |  |  |  |
| **Parental mental illness** | 1.83 | 0.00 | - | 0.72 | 2.38 | 4.44 | 3.64 | 2.04 |
|  |  |  |  |  |  |  |  |  |
| **Sub-optimal maternal bonding** | 18.28 | 9.09 | 8.33 | - | 19.79 | 17.14 | 17.95 | 59.09 |
|  |  |  |  |  |  |  |  |  |
| **Parental physical illness or disability** | 29.36 | 22.22 | 35.71 | 26.81 | - | 15.56 | 25.45 | 36.73 |
|  |  |  |  |  |  |  |  |  |
| **Parental divorce or separation** | 5.50 | 16.67 | 14.29 | 4.35 | 3.33 | - | 0.00 | 10.20 |
|  |  |  |  |  |  |  |  |  |
| **Parental death** | 5.50 | 55.56 | 14.29 | 5.07 | 6.67 | 0.00 | - | 12.24 |
|  |  |  |  |  |  |  |  |  |
| **Maltreatment** | 6.00 | 6.25 | 7.69 | 19.40 | 9.57 | 17.86 | 13.33 | - |

Note: Percentages presented represent the % of participants with the adversity listed in the top row who also experienced each other type of adversity measures.

S 14. Associations of childhood SEP and cumulative psychosocial adversity with methylation age acceleration, restricting analysis to participants with complete data

|  | **ALSPAC 29 year data** | **ALSPAC 47 year data** | **NSHD** |
| --- | --- | --- | --- |
|  | (n = 646) | (n = 610) | (n = 650) |
|  | Mean difference (95% CI) | | |
| **Psychosocial adversity score *** |  |  |  |
| 0 (ref) |  |  |  |
| 1 | 0.26 (-1.23, 0.71) | 0.22 (-0.83, 1.27) | 0.81 (-0.14, 1.77) |
| 2 | -0.96 (-2.15, 0.23) | 0.40 (-0.88, 1.67) | -0.45 (-1.77, 0.86) |
| 3 | -0.26 (-1.42, 0.91) | -0.20 (-1.43, 1.04) | -1.09 (-3.31, 1.12) |
| **Childhood SEP**** | -0.10 (-0.89, 0.69) | -0.52 (-1.37, 0.33) | 0.45 (-0.42, 1.32) |
| * adjusted for childhood SEP | |  |  |
| ** adjusted for psychosocial adversity score | |  |  |

S 15. Associations between the extended cumulative psychosocial adversity score in ALSPAC and methylation age acceleration

|  | **Psychosocial adversity score** | | **29 years** | | **47 years** | |
| --- | --- | --- | --- | --- | --- | --- |
|  |  |  | (n = 989) | | | |
|  |  |  | Mean difference (95% CI) | | | |
|  | Unadjusted | |  | |  | |
|  | 1 | | -0.08 (-0.98, 0.82) | | 0.36 (-0.58, 1.31) | |
|  | 2 | | -0.17 (-1.25, 0.92) | | 0.57 (-0.52, 1.66) | |
|  | 3+ | | -0.25 (-1.15, 0.65) | | 0.01 (-0.94, 0.95) | |
|  | Adjusted for childhood SEP | |  | |  | |
|  | 1 | | -0.08 (-0.99, 0.82) | | 0.36 (-0.59, 1.30) | |
|  | 2 | | -0.18 (-1.28, 0.91) | | 0.56 (-0.54, 1.65) | |
|  | 3+ | | -0.23 (-1.13, 0.67) | | 0.03 (-0.92, 0.97) | |
|  | Additionally adjusted for adulthood SEP | |  | |  | |
|  | 1 | | -0.09 (-0.99, 0.81) | | 0.33 (-0.61, 1.27) | |
|  | 2 | | -0.19 (-1.28, 0.90) | | 0.55 (-0.54, 1.64) | |
|  | 3+ | | -0.22 (-1.13, 0.68) | | 0.05 (-0.90, 0.99) | |
|  | |  | |  | |  |

S 16. Adjusted associations between all types of psychosocial adversity and methylation age acceleration

|  | **ALSPAC 29 year data** | **ALSPAC 47 year data** | | | **NSHD** |
| --- | --- | --- | --- | --- | --- |
|  | (n = 989) | (n = 989) | | | (n = 773) |
|  | Mean difference in methylation age acceleration (years) (95% CI) | | | | |
|  |  | | |  |  |
| **Parent physical illness** |  | | |  |  |
| Adjusted childhood SEP | -0.38 (-1.13, 0.37) | | | 0.09 (-0.70, 0.88) | -0.63 (-1.54, 0.27) |
| Additionally adjusted adult SEP | -0.39 (-1.15, 0.36) | | | 0.05 (-0.74, 0.84) | -0.61 (-1.51, 0.30) |
| **Parental absence** |  | | |  |  |
| Adjusted childhood SEP | 0.26 (-0.65, 1.17) | | | -0.002 (-0.95, 0.95) | 0.75 (-1.91, 3.41) |
| Additionally adjusted adult SEP | 0.27 (-0.64, 1.18) | | | 0.04 (-0.92, 0.99) | 0.89 (-1.77, 3.55) |
| **Childhood physical illness** |  | | |  |  |
| Adjusted childhood SEP | -0.72 (-2.28, 0.83) | | | 0.08 (-1.53, 1.69) | 0.07 (-1.08, 1.22) |
| Additionally adjusted adult SEP | -0.73 (-2.29, 0.83) | | | 0.04 (-1.57, 1.65) | 0.16 (-0.99, 1.31) |
| **Parent mental illness** |  | | |  |  |
| Adjusted childhood SEP | -0.78 (-1.55, -0.02) | | | -0.01 (-0.78, 0.75) | 2.42 (-0.58, 5.43) |
| Additionally adjusted adult SEP | -0.78 (-1.54, -0.02) | | | -0.0001 (-0.77, 0.77) | 2.41 (-0.59, 5.41) |
| **Sub-optimal maternal bonding** |  | | |  |  |
| Adjusted childhood SEP | -0.10 (-1.09, 0.88) | | | -0.13 (-1.10, 0.84) | -0.03 (-1.08, 1.02) |
| Additionally adjusted adult SEP | -0.11 (-1.09, 0.88) | | | -0.16 (-1.13, 0.82) | -0.06 (-1.10, 0.99) |
| **Parental separation** |  | | |  |  |
| Adjusted childhood SEP | 0.32 (-0.68, 1.32) | | | -0.53 (-1.57, 0.51) | -0.98 (-2.69, 0.74) |
| Additionally adjusted adult SEP | 0.34 (-0.66, 1.33) | | | -0.46 (-1.50, 0.59) | -0.85 (-2.57, 0.86) |
| **Parental death** |  | | |  |  |
| Adjusted childhood SEP | 0.73 (-0.86, 2.31) | | | 0.30 (-1.32, 1.92) | -0.27 (-1.83, 1.30) |
| Additionally adjusted adult SEP | 0.74 (-0.85, 2.32) | | | 0.34 (-1.28, 1.96) | -0.17 (-1.73, 1.40) |
| **Maltreatment** |  | | |  |  |
| Adjusted childhood SEP | 0.07 (-0.75, 0.88) | | | 0.14 (-0.75, 1.03) | -1.26 (-2.94, 0.42) |
| Additionally adjusted adult SEP | 0.07 (-0.75, 0.88) | | | 0.15 (-0.74, 1.04) | -1.24 (-2.91, 0.43) |
| **Adoption** |  | |  | | - |
| Adjusted childhood SEP | 0.16 (-2.02, 2.34) | | -0.87 (-3.15, 1.41) | |  |
| Additionally adjusted adult SEP | 0.16 (-2.02, 2.34) | | -0.86 (-3.14, 1.41) | |  |
| **Physical cruelty** |  | |  | | - |
| Adjusted childhood SEP | 0.90 (-0.97, 2.77) | | 0.22 (-1.76, 2.20) | |  |
| Additionally adjusted adult SEP | 0.90 (-0.98, 2.77) | | 0.19 (-1.80, 2.17) | |  |
| **Emotional cruelty** |  | |  | | - |
| Adjusted childhood SEP | -0.27 (-1.50, 0.97) | | -0.31 (-1.59, 0.98) | |  |
| Additionally adjusted adult SEP | -0.27 (-1.50, 0.97) | | -0.30 (-1.60, 0.98) | |  |
| **Physical neglect** |  | |  | | - |
| Adjusted childhood SEP | -0.79 (-3.57, 1.99) | | -2.03 (-5.36, 1.31) | |  |
| Additionally adjusted adult SEP | -0.82 (-3.60, 1.97) | | -2.17 (-5.53, 1.19) | |  |
| **Emotional neglect** |  | |  | | - |
| Adjusted childhood SEP | 0.29 (-0.56, 1.14) | | -0.11 (-1.00, 0.78) | |  |
| Additionally adjusted adult SEP | 0.30 (-0.55, 1.15) | | -0.10 (-0.99, 0.79) | |  |
| **Spent time in care** |  | |  | | - |
| Adjusted childhood SEP | -0.36 (-3.45, 2.74) | | -1.44 (-4.72, 1.84) | |  |
| Additionally adjusted adult SEP | -0.35 (-3.44, 2.74) | | -1.38 (-4.66, 1.89) | |  |
| **Poor family function** |  | |  | | - |
| Adjusted childhood SEP | 0.99 (-0.07, 2.04) | | -0.43 (-1.67, 0.81) | |  |
| Additionally adjusted adult SEP | 1.00 (-0.06, 2.06) | | -0.38 (-1.63, 0.87) | |  |
| **Sexual Abuse** |  | |  | | - |
| Adjusted childhood SEP | 2.77 (0.96, 4.59) | | 3.38 (1.50, 5.25) | |  |
| Additionally adjusted adult SEP | 2.78 (0.96, 4.59) | | 3.41 (1.53, 5.29) | |  |

S 17. Associations for sexual abuse and epigenetic age acceleration in non-imputed data in ALSPAC.

| **Sexual abuse** |  | ***N*** | **Mean difference in methylation age acceleration (years) (95% CI)** |
| --- | --- | --- | --- |
| **ALSPAC 29 year data** |  |  |  |
| Unadjusted |  | 918 | 2.57 (0.72, 4.41) |
| Adjusted childhood SEP |  | 791 | 3.33 (1.24, 5.42) |
| Additionally adjusted adult SEP |  | 788 | 3.35 (1.27, 5.44) |
| **ALSPAC 47 year data** |  |  |  |
| Unadjusted |  | 877 | 3.31 (1.43, 5.19) |
| Adjusted childhood SEP |  | 755 | 3.83 (1.67, 6.01) |
| Additionally adjusted adult SEP |  | 753 | 3.85 (1.68, 6.01) |

S Figure 1. Epigenetic age acceleration distribution for sexual abuse experiences in non-imputed 29 year ALSPAC data

S Figure 2. Epigenetic age acceleration distribution for sexual abuse experiences in non-imputed 47 year ALSPAC data

**References**

1. Parker, G. (1979) Parental characteristics in relation to depressive disorders. *The British Journal of Psychiatry*, 134, 138-47.

2. Muthen, LK, and Muthen, B.O. (1998) Mplus User's Guide. Seventh Edition. Los Angeles, CA: Muthen & Muthen.

3. Tucker, L.R., and Lewis, C. (1973) A reliability coefficient for maximum likelihood factor analysis. *Psychometrika*, 38, 1-10.
